# Acknowledgment to Reviewers of *Vision* in 2021

**DOI:** 10.3390/vision6010009

**Published:** 2022-01-25

**Authors:** Vision Editorial Office

**Affiliations:** MDPI AG, St. Alban-Anlage 66, 4052 Basel, Switzerland

Rigorous peer-reviews are the basis of high-quality academic publishing. Thanks to the great efforts of our reviewers, *Vision* was able to maintain its standards for the high quality of its published papers. Thanks to the contribution of our reviewers, in 2021, the median time to first decision was 27 days and the median time to publication was 60.5 days. The editors would like to extend their gratitude and recognition to the following reviewers for their precious time and dedication, regardless of whether the papers they reviewed were finally published:
Adam ReevesJenni RediferAdriano MagliJenny ReadAlberto ModeneseJoan OlivaAlejandro Martínez-ÁguilaJoana AchaAlessandro MeduriJoanna Dolar-SzczasnyAlessandro RizziJoanna KonopińskaAlessandro SoranzoJoon Mo KimAlexander SchützJulia SongAlgis VingrysJulie HarrisAli BoolaniKaan AkşitAli Mahdavi FardKaren HampsonAlyson J. ChroustKatarzyna HarezlakAndreas KatsanosKay D. BeharryAntonio BerguaKen PfeuferArtyom ZinchenkoKevin DarbyAyelet SapirKowa KoidaBranka SpeharKrzysztof PetelczycBruno RichardLaura KaltwasserCaspar StephaniLaura TascónCatherine M. SuttleLouise O’HareCédrick T. BonnetLubica DudakovaChiara PosarelliLuca BattagliniChloe Callahan-FlintoftLuigi Alberto PiniChristine Anne KiireLyudmila Bel’skayaChristoph Huber-HuberMahalakshmi RamamurthyChristoph MitschManuel PereaChristoph SchankinMarcello ManigliaChristopher Mark HillMark A. GreinerDanial RoshandelMark W. GreenleeDavide BorroniMarla J.S. MickleboroughDe Gaulle I. ChigbuMeaghan CloughDomenico VistoccoMichal S. NowakDonatella UsaiMichele LanzaElia GattoMiguel Ángel Sánchez-TenaEmilija ZdravevaMiguel Martínez GarcíaEvan PalmerMilena RaffiEwa Niechwiej-SzwedoNick Di GirolamoFawzia Bardag-GorceNicole RutaFidel VegaNoémie JaquierFlora GattiParisa GazeraniFrancesco Saverio SorrentinoPatrick CavanaghGeoff ColePaul HibbardGeorgios D. PanosPaul LintonGianluca CoppolaPeter CorcoranGilles LesieurPiotr KanclerzGiovanni BocciaRaffaele OrnelloGiovanni Luca RomanoRalph WeidnerGiulia PurpuraRichard LegrasGuido MaielloRizwanAli NaqviHarold HillRonald HübnerHassane HotaitSergey PetrovHidetaka NomaShane J. HavensHumberto HernandezSławomir CisieckiIkuya MurakamSurya GayetIrene SperandioThomas A. BuseyItiel E. DrorTristan T. HormelJames BrasicVaitsa GiannouliJan P. RöerXinbo LiJanosch Heller



